# Unveiling Evolutionary Path of Nanogenerator Technology: A Novel Method Based on Sentence-BERT

**DOI:** 10.3390/nano12122018

**Published:** 2022-06-11

**Authors:** Huailan Liu, Rui Zhang, Yufei Liu, Cunxiang He

**Affiliations:** 1 School of Mechanical Science and Engineering, Huazhong University of Science and Technology, Wuhan 430074, China; lhlan309@163.com (H.L.); zr2020@hust.edu.cn (R.Z.); hcx@hust.edu.cn (C.H.); 2 Center for Strategic Studies, Chinese Academy of Engineering, Beijing 100088, China

**Keywords:** technology evolutionary path, multi-source data, nanogenerator, text vectorization, theme mining, theme river map

## Abstract

In recent years, nanogenerator technology has developed rapidly with the rise of cloud computing, artificial intelligence, and other fields. Therefore, the quick identification of the evolutionary path of nanogenerator technology from a large amount of data attracts much attention. It is of great significance in grasping technical trends and analyzing technical areas of interest. However, there are some limitations in previous studies. On the one hand, previous research on technological evolution has generally utilized bibliometrics, patent analysis, and citations between patents and papers, ignoring the rich semantic information contained therein; on the other hand, its evolution analysis perspective is single, and it is difficult to obtain accurate results. Therefore, this paper proposes a new framework based on the methods of Sentence-BERT and phrase mining, using multi-source data, such as papers and patents, to unveil the evolutionary path of nanogenerator technology. Firstly, using text vectorization, clustering algorithms, and the phrase mining method, current technical themes of significant interest to researchers can be obtained. Next, this paper correlates the multi-source fusion themes through semantic similarity calculation and demonstrates the multi-dimensional technology evolutionary path by using the “theme river map”. Finally, this paper presents an evolution analysis from the perspective of frontier research and technology research, so as to discover the development focus of nanogenerators and predict the future application prospects of nanogenerator technology.

## 1. Introduction

Nanogenerators are an emerging technology that has attracted a great deal of attention. Nanogenerators are promising for applications in areas including, but not limited to, self-powered systems, mechanical or thermal energy harvesting, and smart wearable devices (SWD) [1,2,3]. At present, the innovation in and research on nanogenerators mainly faces two challenges. The first challenge is that since there are multiple evolutionary paths for nanogenerators, it is difficult to grasp their real development trend. The second challenge is that nanotechnology has many subfields, such as nanomaterials, nanoscale measurements, and nanoscale processing. Nanogenerator technology will cross-penetrate and converge with other nanotechnology sub-fields. The connection between these fields is constantly strengthening and changing. Therefore, it is of great practical significance to determine the development context of nanogenerators, unveil the evolutionary path, and judge the development trends and directions.

The evolutionary path of nanogenerator technology describes the emergence, transition, and demise of different technical themes, which can help researchers understand the history and status of the research field, so as to identify research areas of interest and gaps quickly. Some experts have studied the evolutionary path and trends of nanogenerator technology. These studies are based on expert knowledge and literature reviews, and the research contents involve nanogenerators’ research directions or a sub-field of nanogenerators [4,5,6,7,8]. With the rapid development of nanogenerators, some experts have used quantitative methods, such as bibliometrics, patent citation analysis, technology roadmap, and text mining, to analyze the evolution trend of nanogenerators [9,10]. However, the method of bibliometrics can only be used to conduct simple statistical analysis, and it is difficult to deeply examine technical texts to obtain specific technical descriptions. Patent citation analysis is limited by the type of citation data and ignores the technical knowledge contained in the literature. Furthermore, poor evolution information and biased results result from using single-dimensional data to contain the technology’s evolutionary path. In order to solve the above problems, we consider the mining of the themes of technical texts in papers and patents through text-mining methods. The typical methods include statistics-based keyword extraction methods (such as TF-IDF and RAKE) and theme model methods (such as LDA, ATM), etc. Since the theme model method is not limited by the type of reference data, it can mine most of the data.

This paper proposes a multi-source data-association analysis framework based on the text vectorization method, which improves the traditional theme model method in two respects. On the one hand, this paper uses automatic phrase mining instead of keyword mining, which improves the mining depth of generator information and the interpretability of the results. On the other hand, this paper uses multi-source text information to identify the evolutionary path of nanogenerators. Next, this paper unveils the evolutionary path clearly through the method of theme river map, so as to discover the current development focus of nanogenerators and predict the future application prospects of nanogenerator technology.

## 2. Literature Review

### 2.1. Development of Nanogenerators

With the continuous development of emerging technologies in artificial intelligence, electronic information, and advanced materials, implantable and wearable electronic products have gained traction, such as devices implanted in vivo (pacemakers, neurostimulators), smart watches, glasses, and bracelets [11,12,13,14,15,16,17,18,19]. In practical medical applications, these electronic products can only be powered by an external power source, resulting in their bulky size and high energy consumption, and they need to be replaced regularly. Therefore, these electronic products require the characteristics of scalability and flexibility [20,21], and the development of sustainable forms of the power supply is the core source of competitiveness in the market. In 2006, Professor Zhong-Lin Wang invented the world’s first piezoelectric nanogenerator (PENG) based on a ZnO nanowire array, which generates an electric field by piezoelectric polarization and drives the movement of electrons, converting mechanical energy into electricity [22]. The initial piezoelectric materials are generally ZnO [23,24,25,26,27], lead zirconate titanate (PZT) [28,29,30,31,32,33,34], BaTiO_3_ (BT) [35,36,37,38,39], and polyvinylidene fluoride [40,41,42,43]. Triboelectric nanogenerators (TENG) were first produced in 2011 and were based on the combination of triboelectric electrification and electrostatic induction. Compared with PENGs, TENGs have the advantages of high yield, low cost, simple structural design, and good stability. Currently, TENGs are widely used in various fields due to their excellent performance [44,45,46], such as Shi et al.’s [47] self-powered flexible microfluidic sensor based on triboelectric charging at the liquid–solid interface, which is used for pressure-sensing and finger-movement-monitoring applications. Yi et al. [48] proposed a stretchable rubber-based TENG with a single-electrode mode as a self-powered body-motion sensor.

### 2.2. Technology Evolutionary Path

The technology evolutionary path originated in the 1940s, which can reveal technological evolution. As a powerful presentation of technological development, the technology evolutionary path can track historical development, explore knowledge diffusion, and predict future technological trends [49,50,51,52]. The technology evolutionary path describes the emergence, transition, and demise of technologies, helping technical managers and related researchers to understand the process and the current state of technological development, in order to identify and locate major technologies and technical priorities quickly [53,54,55,56,57,58,59,60,61,62].

In the field of technological evolution research, the research methods mainly fall within the following categories: bibliometrics, social network analysis, and text mining. The common method of early technology evolutionary path research is bibliometrics, which analyzes technological evolution through simple index measurement, co-occurrence analysis, and co-citation analysis. The data sources for these analyses are mainly papers or patent data. Gao, L. et al. [63] proposed a technology life-cycle analysis combining a variety of patent metrics. Co-occurrence analysis is also an important analysis method in bibliometrics, including co-word analysis, proposed by Callon et al. [64], co-author analysis, proposed by Braun et al. [65], and co-citation analysis, proposed by Small [61], among others. The development of bibliometrics is relatively mature, and most of its methods are based on co-occurrence analysis and citation analysis. The principle of this method is relatively simple, and the results can be obtained quickly, but it is difficult to mine the technology evolutionary path directly in this way.

Social network analysis methods are used for the technical mining of the citation information contained in the scientific literature; specific examples of these methods include main path analysis and network topology clustering. Small [66] studied knowledge diffusion through main path analysis, and Kim and Shin [67] identified the main technical paths of HVDC technology through main path analysis. Network topology clustering enables the deeper mining of citation networks and identifies major research communities in the citation network. Chen et al. [68] used the Girvan–Newman clustering algorithm to identify clusters of patent citation networks and found several major technology clusters included in fuel-cell technology, thereby analyzing technological evolution. However, social network analysis ignores important semantic information in the literature data, and the depth of information mining is limited.

At present, the research on technological evolution is based on text mining for technology mining and quantitative analysis. For research data, such as patent papers, this method uses keyword extraction or the theme model to mine text information [69] (including titles, abstracts, claims, etc.) and analyzes the text information based on words or themes [70]. For example, Li et al. used citation analysis to monitor and predict the development trend of nanogenerators, and used the hierarchical Dirichlet Process theme model to extract technical themes [71].

### 2.3. Research on Multi-Source Data and Text Mining

Using papers or patents for technological evolution analysis alone can cause data defects, and the analysis results are often affected by the inherent characteristics of the data, which ultimately affect the accuracy of the analysis. Specifically, papers are focused on the advancement of scientific knowledge, so it is difficult to judge the application of the technology in industry [69]; patents are more inclined to describe the status of technological development but may ignore forward- scientific ideas [69]. In response to the problem of knowledge bias and lack of information caused by single data, some researchers have begun to consider multi-source data analysis to expand the data dimension and analysis perspective. They improve the comprehensiveness of technology evolution analysis by combining these mining data. In general, researchers collect different types of data (including papers, patents, product databases, trade data, news, policy reports, business data, etc.) and combine them with big data fusion and processing methods for technical mining.

In the current research on the technology evolutionary path, finding and presenting thematic information is the key issue, but the results obtained by the theme mining method are keywords, which are difficult to interpret manually. Keyword mining can be further optimized into phrase mining. Compared with technical keywords, technical phrases provide semantic metadata to summarize and describe documents. High-quality phrases contain relatively complete technical information, which is easier for analysts to analyze and can greatly improve the efficiency of technical mining. In the field of phrase mining, TF-IDF is the earliest keyword or phrase extraction algorithm; it ranks phrases according to the frequency of words and inverse document frequency. It considers fewer information extraction factors, so the quality of phrases is usually uncontrollable. KEA is a classic supervised keyword extraction algorithm. It first finds candidate key phrases in the article according to the dictionary, then calculates the phrase feature values and predicts key phrases based on machine learning algorithms [72]. However, KEA is a supervised keyword extraction algorithm that relies on feature computation and has a low degree of automation.

The latest phrase mining research has made great breakthroughs, mainly including a series of phrase-mining algorithms proposed by Han Jiawei et al., such as TopMine, SegPhrase, and AutoPhrase. The previous unigram model (Uni-gram) regards words as the basic units and does not consider contextual meanings, while TopMine’s theme mining of text corpus is carried out in two steps, avoiding the segmentation of words in a phrase. The first step carries out phrase mining for text segmentation, and the second step adds phrase constraints for LDA theme modeling [73]. TopMine is an unsupervised method based on frequency pattern mining and statistical analysis. The SegPhrase algorithm adds labeling work to TopMine, and the quality of the generated phrases is close to the phrase judgment ability of humans [74]. SegPhrase has good scalability and is suitable for large text corpora. In order to avoid manual labeling, Shang et al. [75] proposed the AutoPhrase algorithm. The aim of the AutoPhrase algorithm is to obtain many high-quality phrases from the public knowledge base, and to use these high-quality phrases to generate a large number of positive sample labels. AutoPhrase achieves better performance, further removing the need for additional manual labeling work [75].

## 3. Methods

**Figure 1:**
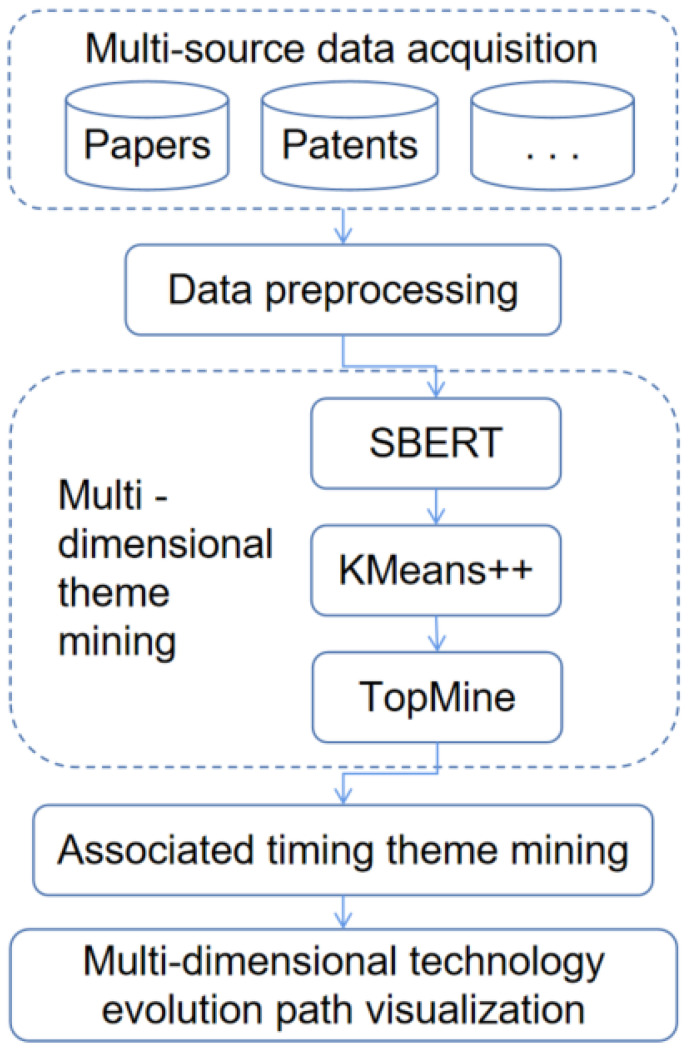
Method and process.

This paper takes multi-source text information mining as the research starting point. First, we present the multi-source data we collected, such as papers and patents, in the field of nanogenerators. Next, we propose a new theme model method to support multi-source data fusion analysis. Finally, we analyze the field from the perspective of frontier science and technological application. We aim to identify the development priorities and future prospects of nanogenerators through the technology evolutionary path. This paper proposes a multi-source data theme modeling method, SKT (Sentence-BERT-KMeans++-TopMine), based on Sentence-BERT and phrase mining. The method flow is shown in Figure 1.

### 3.1. Data

First, we selected the Thomson Reuters database, Web of Science (WOS). By establishing a search strategy, we searched papers based on keywords in the field of nanogenerators. We collected paper data from 2006 to 2022, including the title of the article, citations, abstracts, and other information. Our patent data were then sourced from the Derwent Innovation Index (DII) and Derwent Innovation Platform (DI) databases. By combining the keywords in the field of nanogenerators and the relevant classification numbers to formulate a patent search formula, we collected patent data from 2006 to 2022, including patent names, citation information, times, and abstracts. Eventually, we retrieved 2373 papers and 984 patents.

### 3.2. Theme Modeling Based on Sentence-BERT

The theme modeling of multi-source text data in this paper includes three steps: multi-source text vectorization, document vector clustering to identify themes, and high-quality phrase mining to identify thematic content.

(1) Vectorization of multi-source text documents based on Sentence-BERT algorithm. Sentence-BERT is a Siamese network based on pre-trained BERT. It can obtain document vectors that are sufficiently semantically meaningful. The algorithm performs optimally on multiple semantic textual similarity (STS) benchmark tasks [76]. We used Sentence-BERT for vectorized representation of multi-source text data to achieve unified representation and fusion of multi-source text data in the same semantic vector space.

(2) Identify themes by clustering document vectors with the KMeans++ algorithm. KMeans++ algorithm is one of the commonly used unsupervised clustering algorithms, which can perform clustering tasks on unlabeled datasets. KMeans++ uses the vector distance as the standard for dividing categories. Its clustering process is fast and simple, so it is suitable for clustering of large vector data. In this paper, we use KMeans++ to perform clustering learning on document vector clusters and divide documents into subject categories. We regard one document cluster as one theme.

(3) Mining high-frequency phrases in document clusters as theme content based on TopMine. TopMine is an unsupervised, fully automatic phrase-extraction algorithm proposed by Han Jiawei et al. in 2014 [73]. Based on frequent pattern mining and statistical analysis, it can automatically extract high-quality phrases from a large number of emerging text corpora. This paper uses TopMine to extract key phrases from multi-source document sets, and the extracted phrases are used as the semantic content of theme document clusters, so as to complete the semantic representation of themes.

### 3.3. Unveiling Evolutionary Path Based on Associated Timing Theme

Before the time-series theme analysis, papers and patent data were segmented according to time, that is, divided into several document intervals. Next, we placed the text document into the corresponding time interval according to the year information. Subsequently, each document had three attributes: time segment number, multi-source data type (analysis dimension), and theme number, which were used for multi-dimensional time-series analysis.

Table 1 presents time slice processing of papers and patent data. This resulted in a total of seven time slices. Next, based on these seven time slices, the time-series theme evolution analysis of multi-source data was carried out.

The multi-source time-series theme association has four association dimensions: (1) the association between sub-themes derived from the same type of data; (2) the association between sub-themes derived from different types of data; (3) the association between the same multi-source fusion theme; (4) the association between different multi-source fusion themes. This paper focuses on the association between fusion themes. If the same sub-theme or fusion theme appeared continuously on the timeline, they were associated.

For the association of different fusion themes, we used cosine similarity between mean vectors of document clusters to judge, and the similarity between mean vectors was regarded as the semantic similarity of the fusion themes. The specific process was as follows: 1. Separate documents based on time segment and theme into clusters and obtain vectors of document clusters by index. 2. Calculate the mean vector of the batch of high-dimensional vectors; the mean vector is regarded as the theme semantic vector. 3. Calculate the cosine similarity between each pair of different themes in adjacent time segments, and plot the distribution of cosine similarities 4. Referring to the pre-similarity distribution diagram, set the similarity correlation threshold, and associate the corresponding theme pairs higher than this threshold.

The range of cosine similarity was (0~1). The closer the similarity is to 0, the more similar the themes. The key to the association of different themes is the selection of the similarity threshold. In order to obtain more effective information and less interfering information, the selection of threshold in this study tended to be conservative, so as to make fewer associations and introduce invalid interference information as little as possible, so as to make the final evolution diagram neat and easy to analyze.

### 3.4. Visualization of Multi-Dimensional Technology Evolutionary Path

The technology evolutionary path is suitable for presentation in the visual form of “theme river map”. The primary use of river map is to present the theme evolution of text data. The implementation methods include ThemeRiver and TextFlow [77,78]. TextFlow is an extension of ThemeRiver. It expresses not only the changes in themes over time, but also the splitting and merging of various themes over time. For example, a theme is divided into two, or multiple themes are merged into one, at a certain time. This can help researchers to better intuitively analyze the evolution patterns between themes. In recent years, some researchers have used multi-source data for technical and industrial analysis [79]. This paper is mainly based on TextFlow’s research work on river maps and uses the D3.js language for implementation [80]. Next, we present the design of a visualization scheme for multi-dimensional data fusion theme paths.

**Figure 2:**
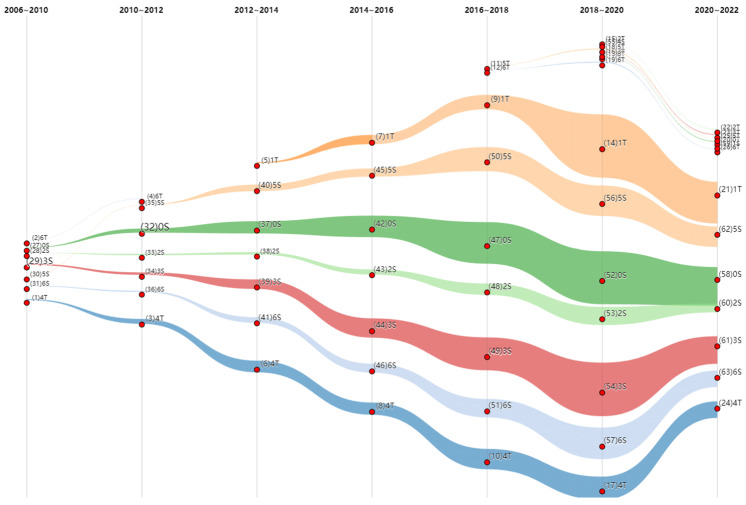
Multi-source theme river map.

Figure 2 presents a thematic river map for unidimensional data. The “rivers” with varying thickness in the figure represent different themes, each of which is distinguished by a different color; each vertical line shows the time segment information, and the “red nodes” on the line represent different themes; the text above the red nodes is brief information about the theme, and the number in brackets is the node number.

**Figure 3:**
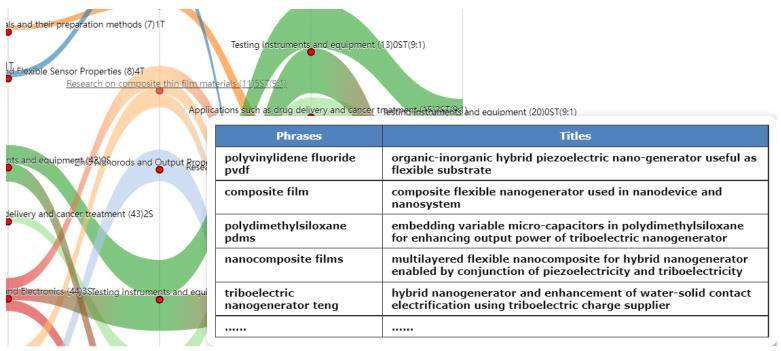
Information presentation of fusion themes.

As shown in Figure 3, the specific information presentation mainly includes the following improvements, which are not only conducive to the presentation of rich details but also to expert interactions or peer discussion: (1) Display of theme type, data source, and theme number. In “(43) 2S”, on the left of Figure 3, 43 is the node number, 2 refers to the theme number, S represents that the theme dimension is in the scientific dimension, and the data source is the WOS paper data. (2) Presentation of the key information of the theme and help the content analysis of the theme. As shown in Figure 3, hovering the mouse near the red node causes the display of detailed information. The figure presents the detailed information of theme 11, including the list of high-frequency theme phrases TOP5 and five randomly selected pieces of document-title information. (3) Display of information on multi-source fusion themes and uses the abbreviation S/T, for Science and Technology, to indicate their attributes. For example, node 11, “theme (11) 5ST (9:1)”, indicates that this is a fusion theme of Science and Technology, and the document ratio is 9:1. In the theme details list, the high-frequency-phrase list and phrase frequency are displayed, and the multi-source text titles of multi-source data sources are marked.

## 4. Experimental Results and Discussion

### 4.1. Multi-Source Theme Identification and Association

According to the theme modeling method proposed in Section 3, we performed joint theme modeling on the papers and patent data corresponding to the scientific and technical layers. As shown in Table 2, according to the high-frequency phrases generated by the time-series theme mining, the seven themes were: testing instruments and equipment (theme 0), research on electrode materials and their preparation methods (theme 1), applications such as drug delivery and cancer treatment (theme 2), wearables and electronics (theme 3), piezoelectric materials and flexible sensor properties (theme 4), research on composite thin-film materials (theme 5), and ZnO nanorods and output properties (theme 6). Next, multi-source time-series theme mining and analysis were carried out to obtain the sub-theme information of each theme corresponding to the different data types and time segments. Finally, we carried out the association of the themes and then identified the theme evolutionary path.

**Figure 4:**
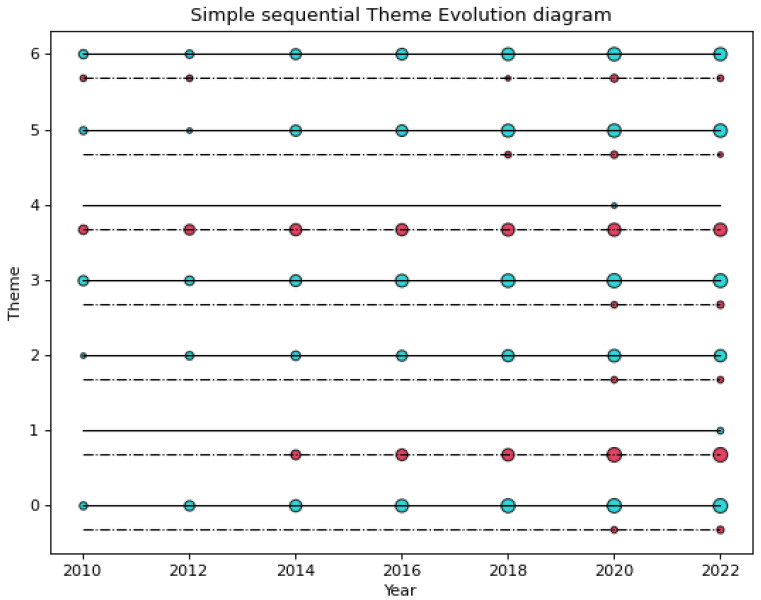
Multi-source data time-series theme-evolution diagram.

We can also use the number of documents to measure the strength of the theme, as shown in Figure 4. This paper draws a schematic diagram of the evolution of multi-source themes with seven themes. The horizontal axis is the time segment from 2006 to 2022, and the vertical axis is the theme number. The themes are divided according to data types, including scientific sub-themes (WOS paper data), circled in blue, and technical sub-themes (DI patent data), circled in red. In the time-series theme-evolution diagram, most of the sub-themes are placed continuously along the timeline (as shown in the blue scientific theme on the timeline of theme 6, at the top of Figure 4), and some sub-themes merge (such as theme 6, where science and technology themes appeared simultaneously in multiple consecutive time segments after 2018).

**Figure 5:**
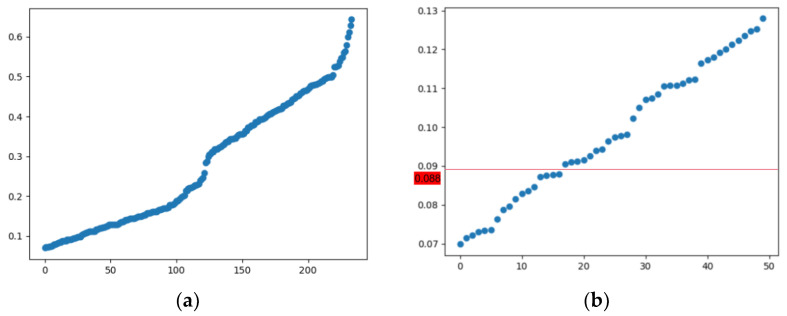
Cosine similarities distribution of adjacent themes: (**a**) Cosine similarities distribution among all adjacent themes; (**b**) distribution of cosine similarities for the top 50.

Next, we used the cosine similarities between the mean vectors to associate different themes, as shown in Figure 5. The cosine similarities were determined in pairs of non-identical themes in adjacent time segments; we then sorted the similarities from small to large and plotted the distribution of the theme cosine similarities. The actual distribution of the similarities was between 0 and 0.7, and the closer the similarity was to 0, the higher the value of the themes’ association.

In order to increase the accuracy of the theme association, we screened out the top 50 cosine similarities. If we observe the similarities distribution in the lower-left corner of Figure 5a, as shown in Figure 5b, it can be seen that the distribution of similarities has a relatively obvious step-shaped change trend. This phenomenon is very helpful for the selection of similarities. Considering the number of associations and the size of the cosine similarities, we selected the second similarity with an obvious step-by-step position as the association threshold, that is, threshold(sim) = 0.088 was selected. This threshold is marked with a red horizontal line in Figure 5b.

After the above theme association experiments, we obtained 57 association relationships, including 17 non-identical pieces of theme association information and 40 of the same pieces of theme association information.

### 4.2. Visualization and Analysis of Multi-Dimensional Technology Evolutionary Path

After the above process, we finally obtained the multi-dimensional technology evolutionary path in the field of nanogenerators based on papers and patent data.

As can be seen from the river map, the intensity of all the themes was weak before 2014, and the research trend in this field was biased towards themes 0 and 3, namely “testing instruments and equipment” and “wearables and electronics”. The use of wearable devices was proposed in 2013 on the basis of basic research on the structure and materials of nanogenerators, and it has also been studied in depth by scholars. The figure shows that composite film materials and zinc oxide nanorod materials have been vigorously developed since 2013. This has accelerated the application of nanotechnology in testing instruments, wearable devices, and electronic products [81]. The results in the figure are consistent with the actual development of nanogenerator technology. In addition, the overall intensity of the river map gradually became stronger after 2014, indicating that nanogenerator technology is developing rapidly.

**Figure 6:**
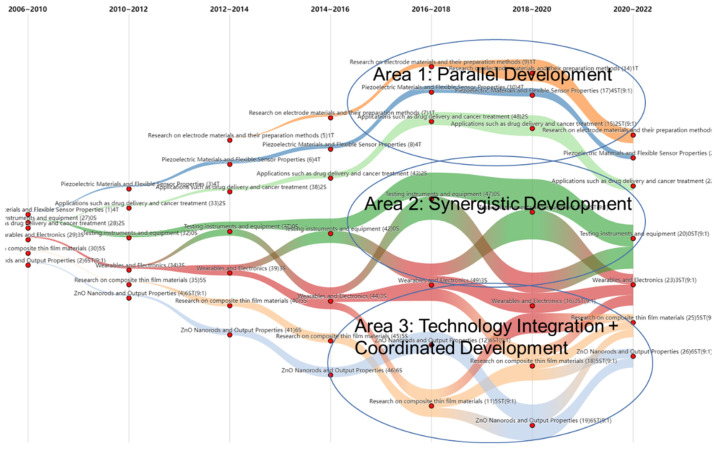
Evolutionary path of nanogenerator technology.

As shown in Figure 6, a significant amount of technology integration and dispersion occurred around 2016–2018, during which opportunities and risks coexisted. We found some laws of development in this phenomenon. From Figure 6, it can be seen that there are three different types of technology evolution across different technologies: parallel development, collaborative development, and technology integration + collaborative development. Among them, theme 1, “research on electrode materials and their preparation methods”, theme 2, “applications such as drug delivery and cancer treatment”, and theme 4 “piezoelectric materials and flexible sensor properties” are under parallel development. These three rivers develop in parallel and rarely intersect. Since 2014, theme 0, “testing instruments and equipment”, and theme 3, “wearables and electronics”, have been developed in synergy, and these two rivers constantly infiltrate each other. Theme 6, “ZnO nanorods and output properties” has been continuously integrated into theme 5, “research on composite thin film materials”, since 2018. There is both technical integration and synergistic development between the two themes.

The technical evolution analysis of multi-source data in the field of nanogenerators reflects and proves the importance of multi-source data to technical analysis. From the river map, we obtained relatively rich technology evolutionary information and analyzed the development status of the nanogenerator field from the perspective of scientific research and technological applications. The improved river map visualization method in this paper is also conducive to the analysis of technological evolution and can improve the utilization of technology mining information.

## 5. Conclusions

This paper proposes a framework for monitoring the evolutionary path of nanogenerator technology based on the Sentence-BERT and phrase-mining methods. We represented technical themes with high-quality phrases through an improved theme evolution modeling approach (SKT) with multi-source text vectorization, which fuses paper and patent text data. The method combines both scientific and technological dimensions to analyze technological evolution in the field of nanogenerators. The experiments showed that our proposed framework is correct and effective.

The study found that TopMine constructs thematic content in the form of phrases, which can enrich thematic connotations and improve thematic interpretability compared with traditional keyword mining. For example, this paper identified themes such as composite thin-film materials, wearable devices, and electronics. The evolutionary path of nanogenerator technology visualized by the river map also revealed much important information. We found that the current development focus of nanogenerator technology is mainly concentrated in several directions, First, the research on electrode materials and their preparation methods and the research on piezoelectric materials and flexible sensor performance are in separate development stages, which indicates that these two research directions are temporary. It is difficult to combine them with other research directions, and they are relatively isolated. Secondly, the research on zinc oxide nanorods and output properties and the research on composite thin film materials are in the stage of technological integration and coordinated development, which shows that these two research directions have undergone mature development. The current research in these two areas is fused and inter-penetrated. The future prospects of nanogenerator technology are mainly concentrated in several directions. For instance, applications such as drug delivery and cancer treatment are in separate development stages, which indicates that they may be in the early stage of application and less combined with other directions, such as detection instruments, equipment, and wearable devices. Furthermore, they are in the stage of coordinated development with electronic products, which shows that these two directions have passed the initial stage of development and are beginning to cooperate with and penetrate each other.

There are some limitations in this study. Specifically, follow-up research could further explore the following aspects: (1) The number of papers and patents in the field of nanogenerators is limited, and it is difficult to fully reflect the advantages of text vectorization and phrase mining. (2) Expert knowledge can be introduced at the multi-source theme identification stage to improve the effectiveness of the analysis of technological evolution.

## Figures and Tables

**Table 1:** Time segment.

Time Segment Number	1	2	3	4	5	6	7
time segment	2006~2010	2010~2012	2012~2014	2014~2016	2016~2018	2018~2020	2020~2022

**Table 2:** Theme modeling results of multi-source fusion texts.

Theme Number	Theme	High-Frequency Theme Phrases
0	Testing instruments and equipment	triboelectric-nanogenerator-based, 54; light-emitting-diodes (LEDs), 33; powered sensor, 32; surface charge density, 32; electronic devices, 30.
1	Research on electrode materials and their preparation methods	friction electric nano generator, 41; nano generator preparation, 30; friction electrode, 29; friction material, 23; electrode layers, 22.
2	Applications such as drug delivery and cancer treatment	triboelectric nanogenerator, 56; drug delivery, 17; cancer therapy, 13; electrical stimulation, 12; drug release, 11.
3	Wearables and electronics	triboelectric nanogenerator teng, 105; wearable electronics, 68; wearable devices, 45; electronic devices, 44; powered sensors, 36.
4	Piezoelectric materials and flexible sensor properties	piezoelectric nano generator, 29; flexible nano generator, 17; high molecular polymer insulating, 16; molecular polymer insulating layer, 16; piezoelectric layer, 16.
5	Research on composite thin-film materials	triboelectric nanogenerator teng, 47; polyvinylidene fluoride pvdf, 34; composite film, 24; polydimethylsiloxane pdms, 22; nanocomposite films, 20.
6	ZnO nanorods and output properties	triboelectric nanogenerator, 57; zno nanorods, 44; output performance, 29; piezoelectric output, 27; zinc oxide zno, 25; electrical output, 22;

## Data Availability

The data presented in this study are available on request from the corresponding author.
